# Fabrication of Quaternized Chitosan Nanoparticles Using Tripolyphosphate/Genipin Dual Cross-Linkers as a Protein Delivery System

**DOI:** 10.3390/polym10111226

**Published:** 2018-11-05

**Authors:** Kuo-Yu Chen, Si-Ying Zeng

**Affiliations:** Department of Chemical and Materials Engineering, National Yunlin University of Science and Technology, Yunlin 64002, Taiwan; M10515035@yuntech.edu.tw

**Keywords:** quaternized chitosan, nanoparticles, bovine serum albumin, tripolyphosphate, genipin, drug release

## Abstract

Various amounts of 2-((acryloyloxy)ethyl)trimethylammonium chloride were grafted onto chitosan (CS) via redox polymerization method to obtain water-soluble quaternized CS (QCS). The QCS nanoparticles loaded with bovine serum albumin (BSA) were then produced by ionic gelation with tripolyphosphate (TPP) and further covalently cross-linked with genipin. The formation of QCS nanoparticles was optimized as a function of monomer grafting yield, QCS/TPP weight ratio, and QCS/genipin weight ratio by Box-Behnken design and response surface methodology. The results showed that QCS nanoparticles prepared with a grafting yield of 50%, QCS/TPP weight ratio of 7.67, and QCS/genipin weight ratio of 60 had a particle size of 193.68 ± 44.92 nm, polydispersity of 0.232, zeta potential of +23.97 mV and BSA encapsulation efficiency of 46.37 ± 2.89%, which were close to the predicted values from mathematical models. In vitro drug release studies at pH 1.2 and pH 7.4 exhibited that the release rate of BSA was significantly decreased and the release period was significantly prolonged after QCS nanoparticles cross-linking with genipin. Therefore, QCS nanoparticles cross-linked with TPP/genipin dual cross-linkers may be a promising protein drug carrier for a prolonged and sustained delivery.

## 1. Introduction

Stimuli-sensitive hydrogels have attracted considerable attention as carriers of drug delivery systems because of the controlled release of drugs upon triggering by external stimuli, including temperature, pH, ionic strength of the surrounding fluid, chemicals, and applied electric/magnetic field [1].

Chitosan (CS) is a natural linear polysaccharide derived by partial deacetylation of chitin. In acidic media (pH < 6.5), CS becomes a cationic polyelectrolyte due to protonation of the amino groups [2]. CS has been widely used in biomedical fields as scaffolds for tissue engineering and wound dressings because of its biodegradability, biocompatibility, nonimmunogenic property, antimicrobial activity, and susceptibility to chemical modifications [3]. CS has also been explored as carriers for mucosal delivery of drugs and bioactive molecules, such as ocular drugs, antipsychotic drugs, peptide, proteins, oligonucleotides, and DNA, due to its mucoadhesive property [4,5,6]. The mucoadhesiveness of CS is due to the ionic interactions between the positively charged protonated amino group of CS and the negatively charged sialic acid and sulfonic acid of the mucus layer [7]. However, CS is only dissolved in an acid solution (pH < 6.5), which may damage cells and bioactive agents [8]. Moreover, CS will be ineffective as an absorption enhancer at physiological pH due to its loss of cationic nature [9]. The poor solubility of CS in physiological media because of strong hydrogen bonding interactions limits its biomedical application [10]. The solubility of CS can be improved remarkably by chemical modification, through covalent addition of hydrophilic groups to CS [11]. The commonly used chemical modifications include cylation, akylation, trimethylation, carboxymethylation, quaternarization, succinylation, and poly(ethylene glycol)ylation [10,12,13,14,15,16].

Previous studies demonstrated that the quaternized derivatives of CS possess high positive charge density and show excellent water solubility over a wide pH range [10]. The cationic charge characteristic of quaternary ammonium moiety is independent of the pH of its surrounding medium [17]. Moreover, quaternized CS could enhance mucosal adhesiveness and facilitate drug delivery across intestinal epithelial cells due to strong electrostatic attraction between the quaternary ammonium groups and the negatively charged endothelial cell membranes [18].

Nanoparticles with mean diameters in the range of hundreds of nanometers have been studied extensively for oral delivery of various drugs due to their greater ability to penetrate epithelial barriers than particles in the micrometer size range [19]. There have been reported several methods to prepare CS nanoparticles, such as emulsion-droplet coalescence, coacervation/precipitation, ionic gelation, self-assembly chemical modification, and reverse micellar method [20]. Among these methods, several investigators used sodium tripolyphosphate (TPP), an anionic cross-linking agent, to prepare CS nanoparticles via reversible electrostatic interactions between the positively charged protonated amino groups of CS and the negatively charged phosphate groups of TPP [21,22]. The process is non-toxic, organic solvent free, easily controllable, mild, and simple [23]. However, relatively weak interactions occur between CS and TPP at low and high pH values.

Various chemical cross-linking agents, such as formaldehyde, glutaraldehyde, glyoxal, epichlorohydrin, and dicarboxylic acids are usually used to increase the stability of CS micro/nanoparticles [24,25]. Moreover, chemical cross-linking could reduce initial burst release and help extend the release period [26]. However, these synthetic cross-linking agents are highly cytotoxic [27]. Genipin, extracted from the fruits of *Gardenia jasminoides* Ellis, is a naturally occurring and low-cytotoxic cross-linking agent [28]. A few researchers used genipin to cross-link CS micro/nanoparticles to improve their physical and chemical stability with good cell compatibility and biocompatibility [29]. Moreover, genipin has been widely used in drug and protein delivery systems to extend the release period [30]. For example, Bhattarai et al. [31] showed that cross-linking poly(ethylene oxide)-grafted CS hydrogels with genipin effectively reduced the initial burst release of bovine serum albumin (BSA) and prolonged release up to 40 days. Yuan et al. [32] indicated that the CS microspheres cross-linked with genipin released BSA more slowly than non-cross-linked microspheres. Karnchanajindanun et al. [33] found that the cumulative release of BSA from genipin-cross-linked CS microspheres decreased as the genipin ratio increased. Nath et al. [34] demonstrated that the release period of bone morphogenetic protein-2 from the CS-hyaluronic acid polyelectrolyte complex cross-linked with genipin could be prolonged over four weeks.

Few studies fabricated CS particles via coupled ionic and chemical co-cross-linking method for biosorbent, biomedical, and drug delivery applications [35,36]. For example, Walke et al. [37] prepared CS microspheres using TPP/vanillin couple cross-linking agents for protein antigens encapsulation. Yang et al. [38] prepared Sonic hedgehog-loaded CS microspheres with TPP/genipin co-cross-linkers and demonstrated the diameter of microspheres increased after cross-linking with genipin. However, studies on the release of drug or protein from quaternized CS nanoparticles cross-linked with TPP/genipin dual cross-linkers have not been reported.

The main focus of the present study was to prepare quaternized CS nanoparticles with TPP/genipin dual cross-linkers and to investigate their controlled release properties. Firstly, quaternary ammonium-containing monomers were grafted into CS by the redox polymerization technique to obtain water-soluble quaternized CS (QCS) with different monomer grafting yields. QCS was characterized by using Fourier transform infrared spectroscopy (FTIR) and wide-angle X-ray diffraction (WAXD). Then, the QCS nanoparticles were prepared by the method of ionic gelation using TPP as a physical cross-linking agent. Furthermore, the initial burst release from nanoparticles was tried to reduce by further chemical cross-linking with genipin. The preparation of QCS nanoparticles was optimized by varying three factors (grafting yield, QCS/TPP weight ratio, and QCS/genipin weight ratio) using Box-Behnken design (BBD) and response surface methodology (RSM). The effects of design factors on the particle size, polydispersity (PDI), and zeta potential of QCS nanoparticles were analyzed by dynamic light scattering (DLS). Moreover, BSA, a globular protein with a molecular weight of 66.5 kDa, was chosen as a model protein drug and encapsulated into the QCS nanoparticles during their preparation. The influence of formulation parameters on the encapsulation efficiency of BSA was also investigated. Finally, the protein release profiles from the QCS nanoparticles were analyzed at pH 1.2 and 7.4 to evaluate their potential as protein carriers.

## 2. Materials and Methods

### 2.1. Materials

Chitosan with deacetylation degree of 75–85% and molecular weight of 50–190 kDa was obtained from Sigma-Aldrich (St. Louis, MO, USA). 2-((Acryloyloxy)ethyl)trimethylammonium chloride (AETMAC, 80 wt % in water) and TPP were also purchased from Sigma-Aldrich. Ammonium persulfate was procured from Showa Chemicals (Tokyo, Japan). Genipin was supplied from Challenge Bioproducts Co. (Yunlin, Taiwan). Bovine serum albumin was purchased from (USBiological, Swampscott, MA, USA). Pierce^®^ BCA protein assay kit was bought from Thermo Fisher Scientific (Rockford, IL, USA). Acetic acid, hydrochloric acid (HCl), and sodium hydroxide (NaOH) were purchased from Katayama Chemical (Osaka, Japan). Acetone and methanol were obtained from Echo Chemical (Miaoli, Taiwan). Phosphate buffer solution (PBS) was supplied from UniRegion Bio-Tech (Hsinchu, Taiwan). All reagents were used as purchased with no further purification. All aqueous solutions were prepared using deionized water.

### 2.2. Synthesis of QCS

**Figure 1:**
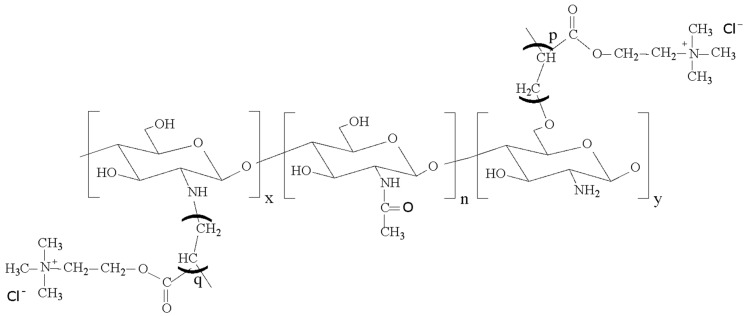
Chemical structure of QCS.

A series of QCS with different contents of quaternary ammonium groups were prepared by graft copolymerization of AETMAC onto CS (Figure 1) using ammonium persulfate as an initiator according to our previous report [39]. Briefly, a 1% (*w*/*v*) aqueous solution of CS was obtained by dissolving CS in 2% (*v*/*v*) acetic aqueous solution using a mechanical stirrer at 80 °C. The solution was purged with nitrogen for 30 min and then 0.06 M ammonium persulfate and various concentrations of AETMAC (0.01, 0.03, and 0.06 M) were added successively dropwise to perform graft polymerization at 80 °C with nitrogen purge. After reaction for 3 h, the reaction solution was cooled to room temperature and poured into excess of acetone with continuous stirring to precipitate the product. The crude product was washed with methanol to remove unreacted AETMAC monomer and poly (2-(acryloyloxy)ethyl)trimethylammonium chloride (PAETMAC) homopolymer. Finally, product was collected by filtration and dried at 60 °C in a vacuum oven. A series of QCS synthesized using 0.01, 0.03, and 0.06 M of AETMAC were named as QCS-30, QCS-40, and QCS-50, respectively. The grafting yield (GY) of QCS was calculated by the gravimetric analysis using the following equation [40]:

(1) $$\;\mathrm{GY}\;\left(\%\right)=\left(\frac{W_{g}-W_{c}}{W_{c}}\right)\times 100\%\;$$

where *W*_g_ and *W*_c_ denote the weight of QCS and CS, respectively.

### 2.3. Preparation of BSA-Loaded QCS Nanoparticles

The QCS nanoparticles were produced by ionic gelation of QCS with TPP anions and simultaneous chemical cross-linking by genipin. Firstly, a 0.2% (*w*/*v*) aqueous solution of QCS was prepared by dissolving QCS with different AETMAC grafting yields in deionized water. The BSA was then dissolved in the QCS solution to give a final concentration of 0.1% (*w*/*v*). After stirring at room temperature for 10 min, TPP/genipin aqueous solutions with different ratios of QCS/TPP and QCS/genipin according to the experimental design were dropped into the above solution using a peristaltic pump over 10 min to in situ gelation of nanoparticles. The weight ratios of QCS/TPP were 5:1, 10:1, and 15:1, while those of QCS/genipin were 20:1, 40:1, and 60:1. The mixed solution was kept stirred at room temperature for 30 min. The cross-linking reaction was further carried out at 35 °C for 3 h with continuous stirring. Subsequently, the dispersion was centrifuged at 10,000 rpm for 20 min to obtain BSA-loaded QCS nanoparticles. The obtained nanoparticles were washed gently with deionized water to remove residual TPP and genipin and unencapsulated BSA from the nanoparticle surface. Finally, the nanoparticles were isolated by centrifugation, resuspended in test medium, and used for further characterization.

### 2.4. Experimental Design for RSM

Design-Expert^®^ software (Stat-Ease Inc., Minneapolis, MN, USA) was used to design the experiment, randomize the runs, and optimize the formulation for preparing nanoparticles. A BBD with three factors at three levels was selected to investigate the influence of three different formulation variables on the properties of QCS nanoparticles (Table 1). The factors studied were AETMAC grafting yield (*A*) and weight ratios of QCS/TPP (*B*) and QCS/genipin (*C*). The design consisted of 15 runs, including 3 replicated center points and 12 axial points. The particle size (*Y*_1_), PDI (*Y*_2_), zeta potential (*Y*_3_), and BSA encapsulation efficiency (*Y*_4_) of the nanoparticles were taken as response parameters. All the responses were fitted to linear, second order, and quadratic models. The best-fit model for each response variable was generated by the software. The suitable models include linear, second order, and quadratic models. A quadratic polynomial model was given as follows [41]:

(2) $$\;Y_{i}=\beta_{0}+\beta_{1}A+\beta_{2}B+\beta_{3}C+\beta_{12}AB+\beta_{13}AC+\beta_{23}BC+\beta_{11}A^{2}+\beta_{22}B^{2}+\beta_{33}C^{2}\;$$

where *Y*_i_ represents the measured response associated with each factor level combination; $\beta_{0}$ is an intercept; *β*_1_, *β*_2_, and *β*_3_ are the linear regression coefficients calculated from the observed experimental values of Y; *β*_12_, *β*_13_, and *β*_23_ are the interaction coefficients between the three factors; *β*_11_, *β*_22_, and *β*_33_ are quadratic coefficients; and *A*, *B*, and *C* are the coded levels of independent variables.

Three dimensional response surface plots were constructed to visualize the relationships between the responses and the formulation parameters. The optimization was performed using the desirability function with the following criteria: (a) particle size below 200 nm; (b) PDI below 0.3; and (c) maximization of encapsulation efficiency (Table 1). The experimental results were compared with the predicted values of the model to evaluate the accuracy of the model.

### 2.5. Characterizations

Infrared spectra of dry CS and QCS in the wavelength range of 4000–500 cm^−1^ were obtained with a FTIR (FTS-40, Bio-Rad, Hercules, CA, USA) using KBr discs. The spectra were recorded in transmission mode at resolution of 2 cm^−1^ and with 32 scanning.

The WAXD patterns of CS and QCS were obtained using a Rigaku MiniflexII X-ray diffractometer (Tokyo, Japan) operating with Cu-*K*α radiation at a scan rate of 2° min^−1^ in the 2θ range of 2–50°.

The water solubility of CS and QCS at pH 6.0 and 7.0 was evaluated by a turbidity assay as previously reported with slight modification [42]. Briefly, 2 mg/mL aqueous solution of CS and QCS were obtained by dissolving CS and QCS in 1% (*v*/*v*) acetic aqueous solution, respectively. One M sodium hydroxide solution was slowly added to adjust the pH value of the solution to 6.0 and 7.0. The transmittance of the solution was recorded on a visible spectrophotometer (SP-830 Plus, Metertech, Taipei, Taiwan) at 600 nm.

The particle size, size distribution (PDI), and zeta potential of QCS nanoparticles were measured by NanoPlus zeta/nano particle analyzer (Micromeritics Instrument Co., Norcross, GA, USA).

### 2.6. In Vitro BSA Release

#### 2.6.1. BSA Encapsulation Efficiency

The BSA encapsulation efficiency of QCS nanoparticles was determined as previously described with modifications [43]. The actual BSA loading amount was calculated through an indirect method by the difference between the initial amount of BSA dissolved in QCS solution and the amount of BSA remaining in the supernatant. Briefly, 25 μL of the supernatant obtained from centrifugation at 10,000 rpm for 20 min was mixed with 200 μL of BCA protein assay reagent. After incubating at 37 °C for 30 min, the concentration of free BSA in the supernatant was measured using a microplate reader (800TS, BioTek Instruments, Inc., Winooski, VT, USA) at a wavelength of 570 nm. A calibration curve was established with measuring the absorbance of the known BSA concentration. The supernatant of non-loaded QCS nanoparticle suspension was used as a blank. The BSA encapsulation efficiency (EE, %) was calculated using the following equation,

(3) $$\;\mathrm{EE}\;\left(\%\right)=\left(\frac{W_{0}-W_{a}}{W_{0}}\right)\times 100\%\;$$

where *W*_0_ is the initial amount of BSA added during preparation of QCS nanoparticles and *W*_a_ is the free amount of BSA in the supernatant.

#### 2.6.2. Cumulative BSA Release

In vitro release profiles of BSA from QCS nanoparticles were investigated in simulated gastric fluid (0.1 N HCI, pH 1.2) and simulated intestinal fluid (PBS, pH 7.4); 4.5 mg of freshly prepared nanoparticles were dispersed in 10 mL of release medium. After being shaken at 60 rpm at 37 °C, 0.5 mL of release medium was collected at predetermined time points and replaced with an equal volume of fresh medium immediately. The sample was centrifuged at 10,000 rpm for 20 min. The amount of released BSA in the supernatant was measured as described above. The cumulative BSA release (CBR, %) of each sample was calculated according to the formula,

(4) $$\;\mathrm{CBR}\;\left(\%\right)=\left(\frac{W_{t}}{W_{\infty }}\right)\times 100\%\;$$

where *W*_t_ is the cumulative amount of BSA released from the nanoparticles at a given time *t* and *W*_∞_ is the amount of BSA initially loaded in the nanoparticles. All drug release experiments were performed in triplicate and average values were taken.

### 2.7. Statistical Analysis

Statistical analyses were performed using Student’s *t*-test or ANOVA, followed by Fisher’s LSD post-hoc multiple comparisons. The level of statistical significance was set at *p* < 0.05.

## 3. Results and Discussion

### 3.1. QCS Characteristics

The synthesis of three graft copolymers based on CS backbone and PAETMAC side chains was performed by varying the AETMAC concentration at constant concentration of CS and initiator. The chemical composition and crystal structure of QCS were examined by FTIR and WAXD analyses.

**Figure 2:**
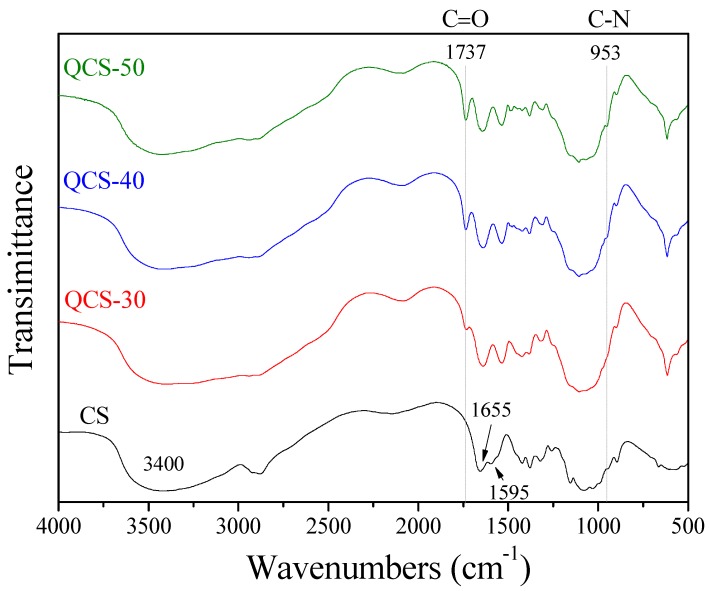
FTIR spectra of CS and QCS.

The FTIR transmittance spectra of CS and QCS are presented in Figure 2. A characteristic strong and broad band appeared at around 3400 cm^−1^, corresponding to O–H and N–H stretching vibrations of hydroxyl and amino groups. The band for the C=O stretching vibration of amide I groups was located at around 1655 cm^−1^. The N–H bending and C–N stretching vibration of amide II appeared at around 1595 cm^−1^. In comparison of the FTIR spectra of QCS and CS, QCS exhibited two additional characteristic absorption bands at 1737 cm^−1^ and 953 cm^−1^, which could be attributed to the C=O stretching vibration of ester groups and the C–N stretching vibration of quaternary ammonium groups of AETMAC [44]. The FTIR data demonstrated that AETMAC was successfully grafted onto the CS. Moreover, the intensities of the absorption bands at 1737 cm^−1^ and 953 cm^−1^ increased with increasing concentration of AETMAC in the reaction mixture, indicating the increase in AETMAC content in QCS.

Furthermore, the grafting yield of AETMAC on CS was estimated by the gravimetric analysis. It was found that the grafting yields of QCS-30, QCS-40, and QCS-50 were approximately 31.8%, 39.9%, and 49.8%, respectively, indicating that the grafting yield increased with the monomer concentration.

**Figure 3:**
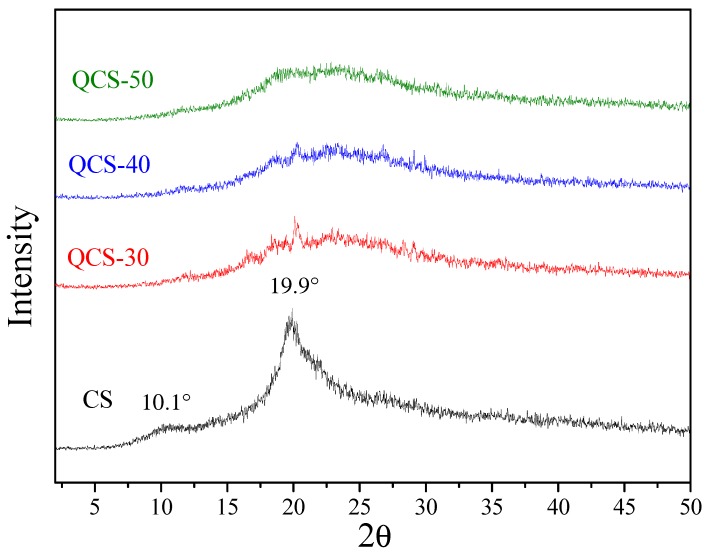
WAXD spectra of CS and QCS.

The X-ray diffraction patterns of CS and QCS with different grafting yields are illustrated in Figure 3. CS had two characteristic crystalline peaks at 2θ = 10.1° and 19.9° corresponding to (020) and (110) crystallographic planes, respectively [45]. In the diffraction pattern of QCS, the peak in the vicinity of 10.1° seems to be disappeared, while the intensity of the peak at 19.9° sharply decreased and broadened with increasing concentration of AETMAC, demonstrating that crystallinity of CS decreased after grafting of PAETMAC. It is seen that the shape of WAXD pattern of QCS-50 was broad and flat, suggesting that it was almost amorphous. This may be ascribed to the introduction of bulky side chains, PAETMAC, onto the CS backbone, which greatly reduced intermolecular and intramolecular hydrogen bonding interactions of CS, resulting in the decrease of crystallinity. Accordingly, the water solubility of CS would be significantly improved through grafting of hydrophilic PAETMAC on CS chains [44].

Table 2 presents the water solubility of CS and QCS at pH 6.0 and 7.0. CS and QCS solutions had a high transmittance value (close to 100%) at pH 6.0, indicating that they were highly soluble in water. At pH = 7.0, the transmittance of CS solution dropped sharply, while that of QCS solutions decreased by a smaller extent. Moreover, the transmittance increased with the increase of grafting yield. These results suggest that the introduction of plenty of hydrophilic quaternary amino groups and bulky side chains to CS are effective means for improving water solubility of CS at neutral pH.

From the FTIR, WAXD, and water solubility results, it can be concluded that AETMAC monomers were successfully grafted onto CS backbone, and the grafting yield increased with increasing concentration of AETMAC.

### 3.2. Design of Experiment

**Figure 4:**
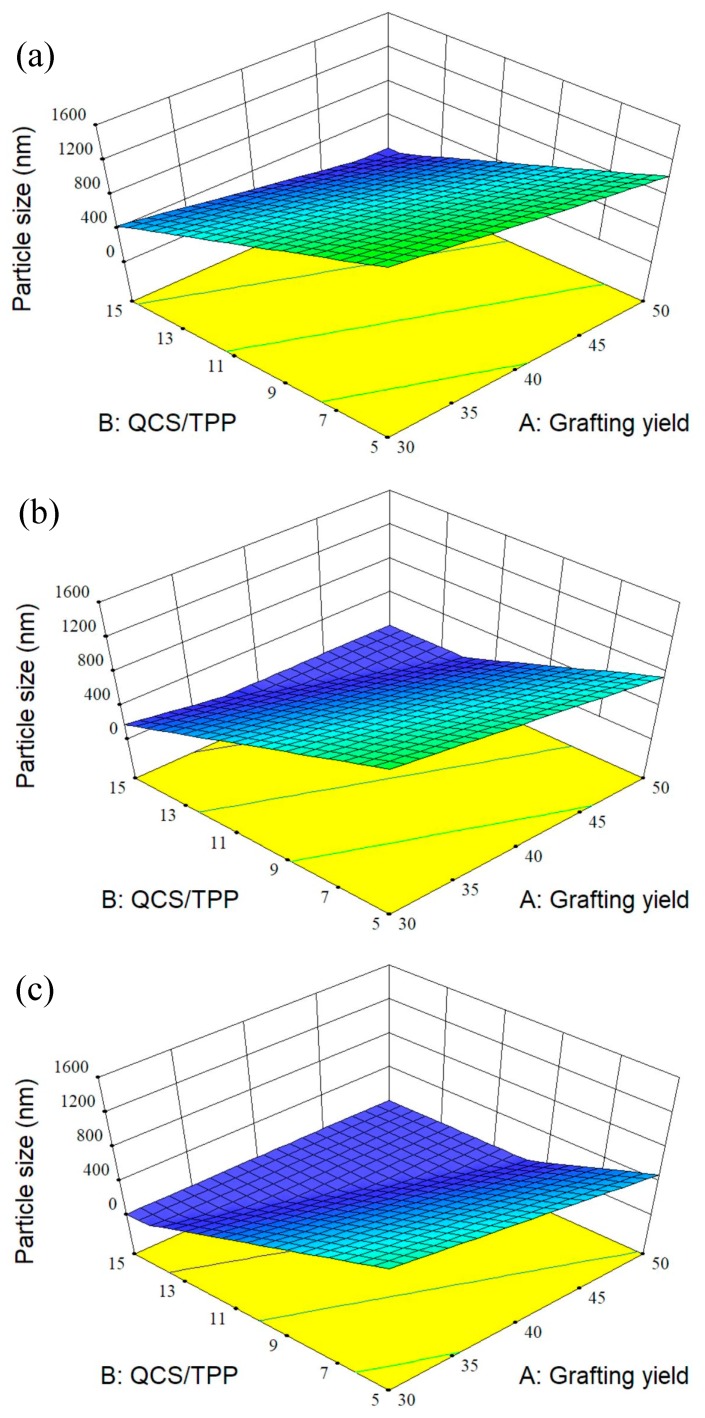
3D response surface plots showing the effects of grafting yield and weight ratio of QCS/TPP at different weight ratios of QCS/genipin of (**a**) 20/1, (**b**) 40/1, and (**c**) 60/1 on particle size.

**Figure 5:**
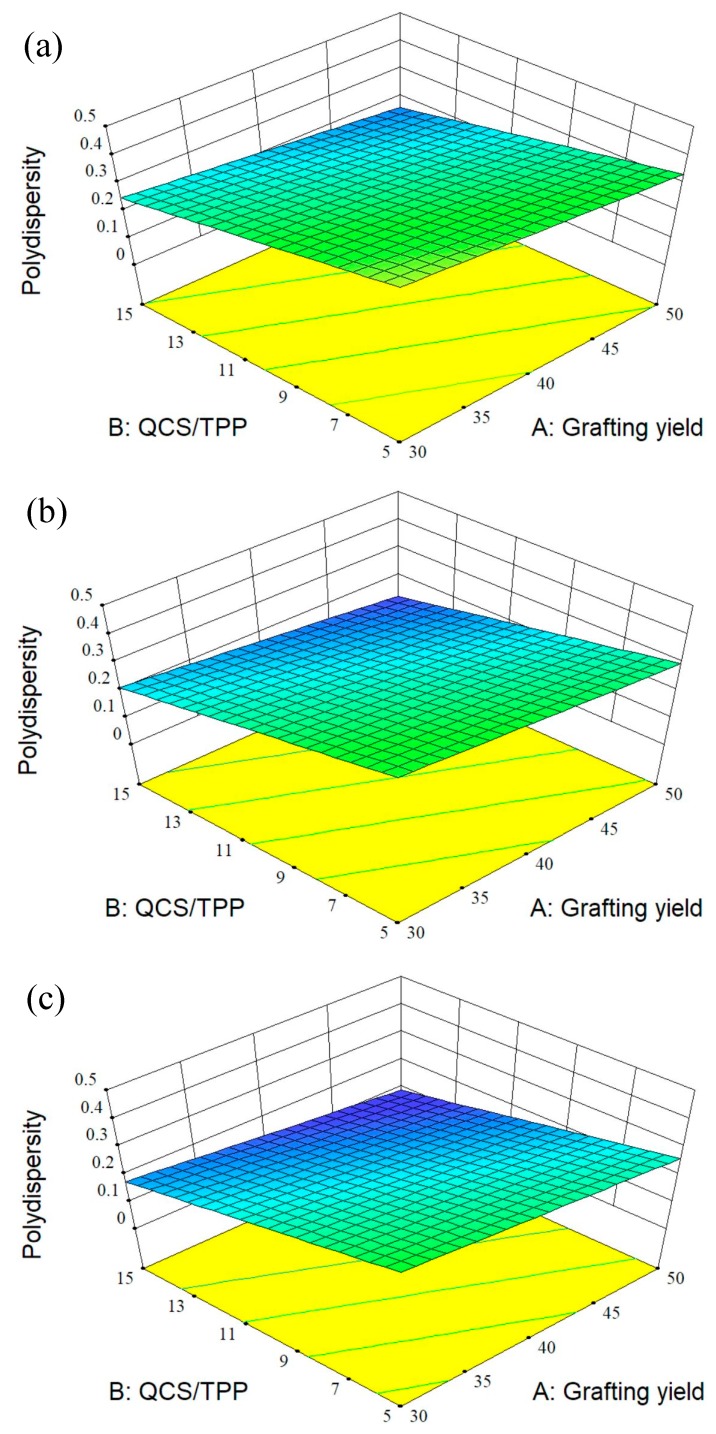
3D response surface plots showing the effects of grafting yield and weight ratio of QCS/TPP at different weight ratios of QCS/genipin of (**a**) 20/1, (**b**) 40/1, and (**c**) 60/1 on PDI.

**Figure 6:**
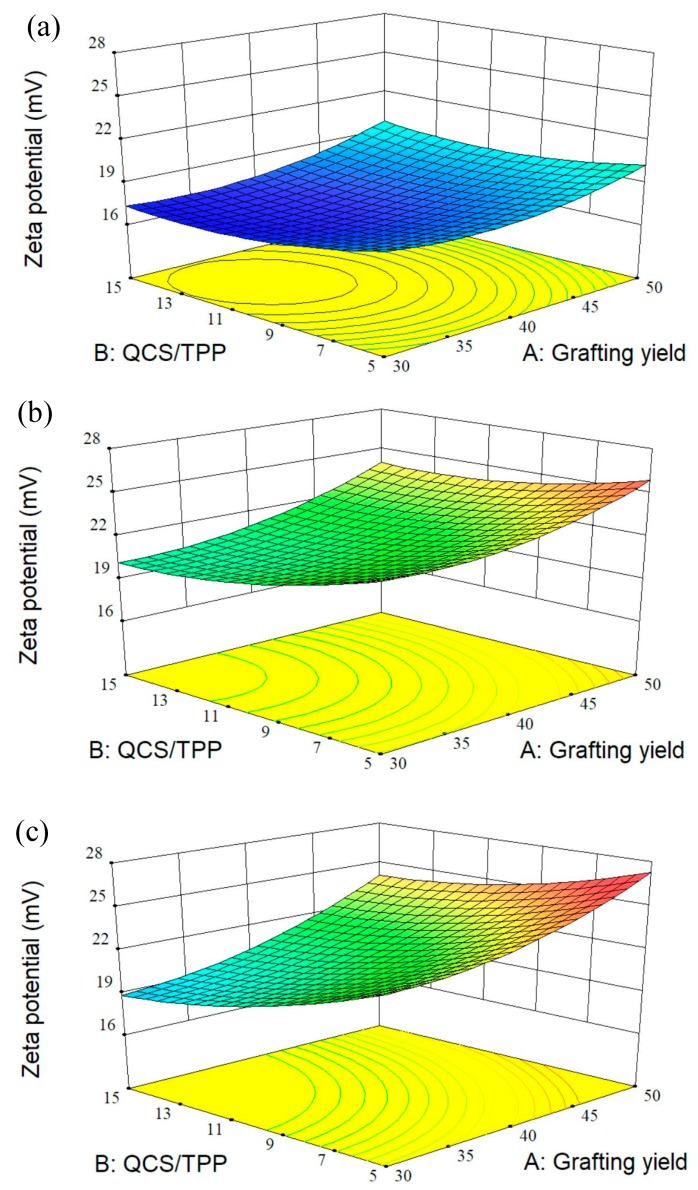
3D response surface plots showing the effects of grafting yield and weight ratio of QCS/TPP at different weight ratios of QCS/genipin of (**a**) 20/1, (**b**) 40/1, and (**c**) 60/1 on zeta potential.

**Figure 7:**
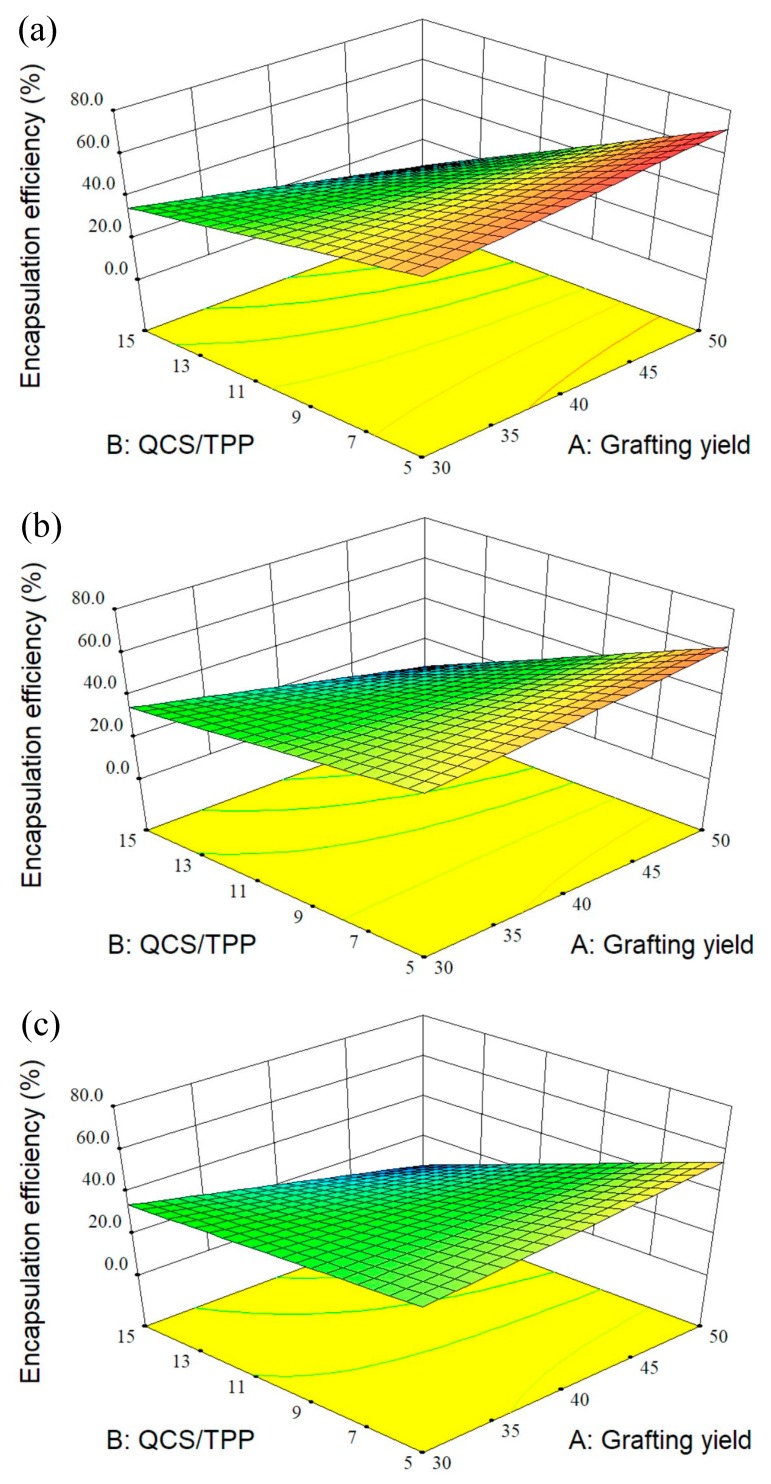
3D response surface plots showing the effects of grafting yield and weight ratio of QCS/TPP at different weight ratios of QCS/genipin of (**a**) 20/1, (**b**) 40/1, and (**c**) 60/1 on encapsulation efficiency.

A few investigations used design of experiment to optimize the conditions for fabrication of CS particles as drug carriers [24,46,47,48]. RSM is a set of experimental design techniques used to obtain the optimal condition with minimum experiments. BBD, one of the most commonly used RSM techniques, requires fewer design points than other response surface designs [49]. This study used BBD to optimize the preparation process of QCS nanoparticles and to investigate the effects of three process variables on the properties of QCS nanoparticles which includes particle size, PDI, zeta potential, and encapsulation efficiency. The relationship between three independent variables and each response was demonstrated by the mathematical models. It was found that the linear models were best-fitted for both particle size and PDI, two-factor interaction (2FI) model for encapsulation efficiency and quadratic model for zeta potential. Further, the 3D response surface curves were plotted by altering grafting yield and QCS/TPP weight ratio in the experimental range and keeping QCS/genipin weight ratio constant (Figure 4, Figure 5, Figure 6 and Figure 7).

#### 3.2.1. Effect of Variables on the Size of QCS Particles

The particle size and distribution are important characters for the efficiency of pharmaceutical formulations. By varying the AETMAC grafting yield, QCS/TPP weight ratio and QCS/genipin weight ratio, QCS particles were obtained with sizes and PDI in the range of 146.65 to 2519.73 nm and 0.125 to 0.571, respectively (Table 3). The linear equations for particle size (*Y*_1_) and PDI (*Y*_2_) are shown below:

(5) $$\;Y_{1}=471.92-257.95A-550.12B-270.21C\;$$

(6) $$\;Y_{2}=0.26-0.045A-0.087B-0.036C\;$$

Negative coefficient of all the factors for *Y*_1_ and *Y*_2_ denotes that the particle size and PDI decreased with increasing grafting yield, QCS/TPP weight ratio, and QCS/genipin weight ratio. The smaller particle size of QCS prepared with higher grafting yield could be due to the stronger electrostatic interactions occurring between the QCS and TPP. Moreover, higher positive charge density could prevent particle aggregation. Therefore, QCS particles with higher grafting yield have both smaller particle size and smaller PDI (Table 3). The increase of QCS/TPP weight ratio and QCS/genipin weight ratio led to the decrease in particle size, which might be attributed to the decreased cross-linking degree of QCS [46]. A similar result has been reported by other researchers. Moreover, Hu et al. [50] also found that the particle size decreased linearly with the increasing CS/TPP weight ratio because the superfluous TPP would link the monoparticles to form larger particles.

#### 3.2.2. Effect of Variables on the Zeta potential of QCS Particles

Zeta potential not only reflects the surface electric potential of particles in solution but also the colloidal stability. In general, the greater the zeta potential, the better stability of the particles in suspension. As shown in Table 3, all the particle formulations had positive zeta potential values ranging from 17.10 to 26.02 mv, indicating the formulation of positively charged QCS particles. The zeta potential of QCS particles could be fitted by the following second-order polynomial quadratic equation:

(7) $$\begin{array}{c} \;Y_{3}=21.50+1.63A-1.21B+2.13C+0.26AB+0.70AC-0.67BC+1.01A^{2}\\ +0.78B^{2}-2.08C^{2}\; \end{array}$$

It can be seen from Figure 6 that the zeta potential values increased with increasing grafting yield due to higher positive charge density of QCS with higher quaternary ammonium groups. As mentioned above, the higher positive charges on the surfaces of particles would result in smaller particle size and narrower PDI. Further, the QCS/genipin weight ratio had a positive effect on zeta potential. The decrease of QCS/genipin weight ratio resulted in the decrease of the zeta potential, which might be attributed to the occurrence of cross-linking reaction between genipin and amino groups on QCS chain. The decrease in the amount of residual amine groups of QCS would decrease the surface charge of particles. Zhang et al. [51] also found that the genipin cross-linked CS microspheres had a lower zeta potential value compared to those without cross-linked with genipin, due to the reaction between genipin and part of the amine groups of CS.

#### 3.2.3. Effect of Variables on the Encapsulation Efficiency of BSA

The BSA entrapment efficiency of QCS particles for 15 runs ranged from 2.23 to 67.76% (Table 3). The 2FI equation for entrapment efficiency (*Y*_4_) is as follows:

(8) $$\;Y_{4}=38.78-4.22A-18.22B-4.31C-10.40AB-0.30AC+3.68BC\;$$

This equation suggests the negative effects caused by increases in grafting yield, QCS/TPP weight ratio, and QCS/genipin weight ratio on encapsulation efficiency. The decrease of QCS/TPP weight ratio and QCS/genipin weight ratio resulted in the increase of the encapsulation efficiency, which might be related to the increase in the cross-linking degree of QCS [20,52]. The effect of varying grafting yield and QCS/TPP weight ratio on encapsulation efficiency was examined when QCS/genipin weight ratio was kept constant (Figure 7). When QCS particles were prepared with higher grafting yield and lower weight ratio of QCS/TPP, a higher encapsulation efficiency was obtained which could be due to the stronger electrostatic interactions occurring between QCS and TPP. However, the encapsulation efficiency obviously decreased with the grafting yield when the QCS/TPP weight ratio was high, indicating that the amount of TPP was not enough to produce densely cross-linked networks with QCS. Therefore, a higher BSA entrapment efficiency could be obtained when QCS had high grafting yield and the weight ratios of QCS/TPP and QCS/genipin were low.

#### 3.2.4. Optimization and Validation

**Figure 8:**
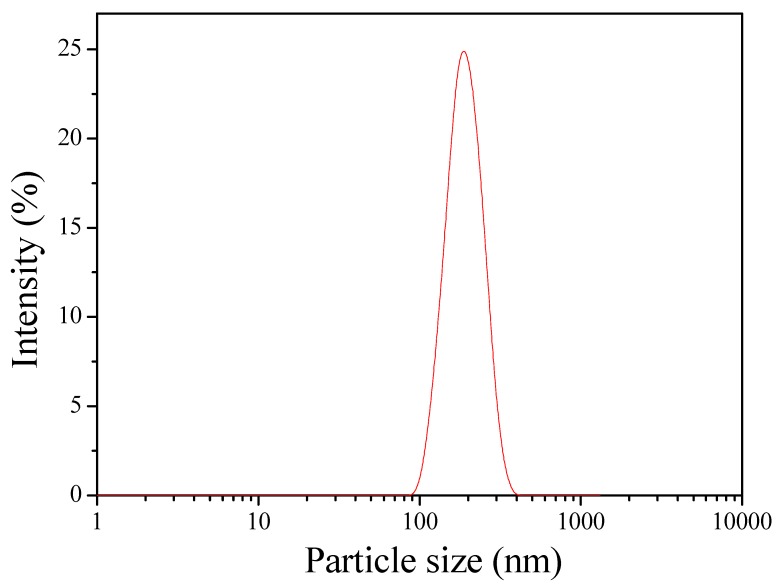
Particle size distribution of the optimized formulation.

The optimum formulation of QCS nanoparticles was conducted using numerical optimization approach based on desirability function. The desirable particle size and PDI were set to be under 200 nm and 0.3, respectively. The encapsulation efficiency was set to approach maximum to ensure effective encapsulation of BSA. The compositions for optimized formulation were 50% grafting yield, 7.67:1 QCS/TPP weight ratio, and 60:1 QCS/genipin weight ratio, respectively. Bikiaris et al. [53,54] found that CS nanoparticles had the smallest particle size when the CS/TPP ratio was 4:1 or 5:1. However, QCS had more positive groups than CS, therefore it needed more TPP to cross-link QCS. The QCS nanoparticles were further prepared with the above optimized formula to confirm the validation of RSM. The particle size and size distribution curve of optimized formulation are shown in Figure 8. The average particle size, PDI, zeta potential, and encapsulation efficiency were found to be 193.68 nm, 0.232, +23.97 mV, and 46.37%, respectively (Table 4). The experimental measurements were quite close to the predicted values from mathematical models. Therefore, the model was adequate for the predication of the selected response.

### 3.3. BSA Release Prolife

**Figure 9:**
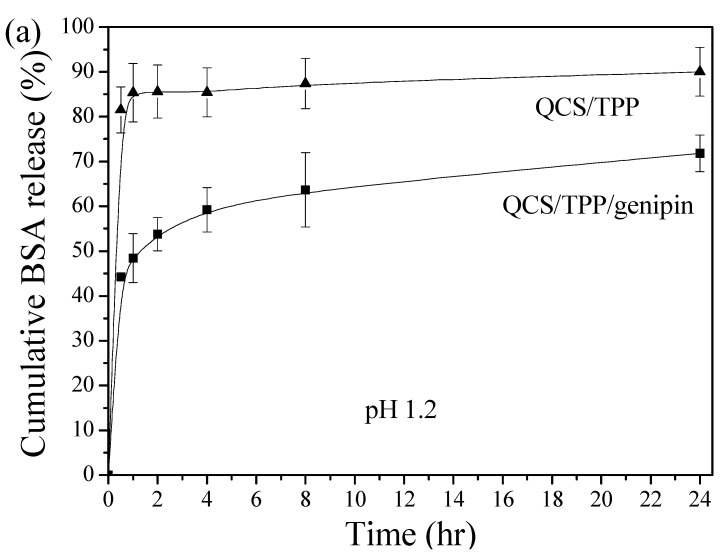

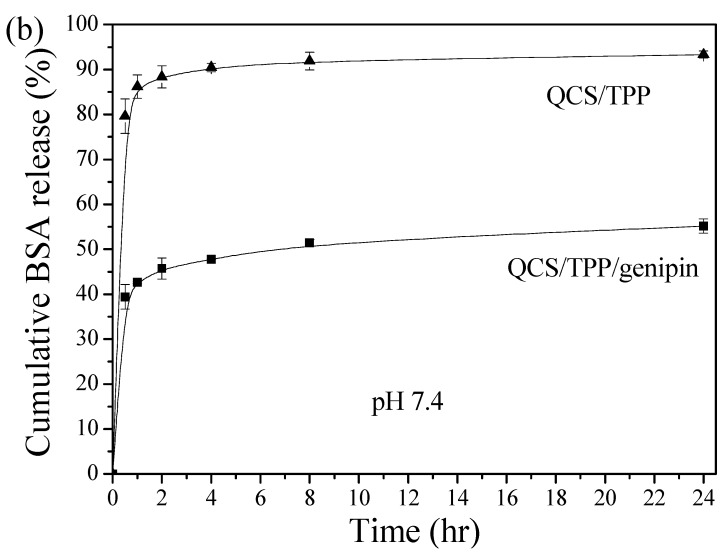
Release profiles of BSA from QCS nanoparticles cross-linked with (QCS/TPP/genipin; ■) or without (QCS/TPP; ▲) genipin at pH of (**a**) 1.2 and (**b**) 7.4 at 37 °C.

In order to assess the feasibility of QCS nanoparticles as drug carriers for therapeutic proteins, the in vitro release of BSA from optimized QCS nanoparticles (QCS/TPP/genipin) was measured in both 0.1 N HCl (pH 1.2, simulated gastric fluid) and PBS (pH 7.4, simulated intestinal fluid) at 37 °C. The QCS nanoparticles cross-linked only with TPP without the presence of genipin (QCS/TPP) were also prepared to investigate the effect of genipin cross-linking on the BSA release. The cumulative release profiles of BSA from the prepared nanoparticles at pH 1.2 and pH 7.4 are presented in Figure 9a,b, respectively. An initial burst release was observed for both QCS/TPP/genipin and QCS/TPP. Most of the encapsulated BSA (85.4% at pH 1.2 and 86.2% at pH 7.4) were released from QCS/TPP within 1 h, suggesting that QCS/TPP was not stable in the release medium. On the contrary, the initial burst release within the first hour was much less pronounced (48.0% at pH 1.2 and 42.6% at pH 7.4) for QCS/TPP/genipin. The initial burst release might be ascribed to the BSA desorption from the surface of the nanoparticles. The much lower burst release from QCS/TPP/genipin suggests that more BSA molecules were encapsulated inside the matrix of QCS/TPP/genipin than in the matrix of QCS/TPP. As indicated above, the encapsulation efficiency was increased with the decrease of QCS/genipin weight ratio. The increase of genipin content led to the increase in the cross-linking degree of QCS, which would lower the degree of burst release. Moreover, QCS/TPP/genipin exhibited a sustained release of BSA at pH 1.2 and 7.4 within 24 h. After 4 h and 24 h, the cumulative BSA release at pH 1.2 was 59.2% and 71.8%, respectively, whereas that at pH 7.4 was 47.8% and 55.2%, respectively. A sustained release of BSA could be attributed to the diffusion of BSA molecules through the polymer matrix and/or erosion of the nanoparticles. These results are consistent with other previous studies that have shown that cross-linking CS hydrogels and microspheres with genipin could effectively reduce the initial burst release of BSA and prolonged release period [31,32,33]. Huang et al. [55] demonstrated that genipin-cross-linking could greatly improve the compactness and regularity of network structure of the O-carboxymethyl chitosan-gum Arabic coacervates. Therefore, the stability of the coacervates could be enhanced in the simulated gastric solution and the BSA release could be reduced in simulated gastrointestinal fluids. In this study, QCS/TPP/genipin demonstrated a superior sustained-release characteristics compared with QCS/TPP, suggesting that the cross-linking between QCS and genipin could effectively increase the stability of nanoparticles in the release medium. Consequently, the protein delivery was greatly reduced and the release period was significantly prolonged.

To investigate the release mechanism of BSA from optimized QCS/TPP/genipin nanoparticles, the release data were fitted in a Korsmeyer–Peppas model [56]:

(9) $$\;\ln\left(\frac{M_{t}}{M_{\infty }}\right)=n\ln t+\ln k\;$$

where *M*_t_ and *M*_∞_ are the amount of drug released in a given time *t* and equilibrium time, respectively. *k* is the release rate constant. *n* is the release exponent, which suggests the release mechanism of drug and depends on the shape of the matrix tested. The *n* values are obtained from the slope of the plot of ln (*M*_t_/*M*_∞_) versus ln *t*. For the case of spherical matrices, *n* ≤ 0.43 indicates Fickian diffusion-controlled release (case I transport). When 0.43 < *n* < 0.85, drug release follows non-Fickian release (anomalous transport). If *n* ≥ 0.85, it indicates relaxation-controlled release (case II transport). The goodness-of-fit of the model was assessed by comparing the coefficient of determination (*R*^2^) using Microsoft Excel software.

The coefficients of determination obtained from fitting experimental results of BSA released from QCS/TPP/genipin nanoparticles at pH 1.2 and 7.4 were 0.9948 and 0.9903, respectively, indicating that release data were well fitted by the Korsmeyer–Peppas model. The exponent *n* values for the release of BSA at pH 1.2 and 7.4 were 0.1262 and 0.0866, respectively, which were both less than 0.43. Thus, Fickian diffusion was the main release mechanism for QCS/TPP/genipin nanoparticles in the first day.

## 4. Conclusions

In this study, water-soluble quaternized CS with different monomer grafting yields were synthesized using the redox-graft polymerization method. The QCA nanoparticles were then prepared by ionic gelation with TPP and chemical cross-linking with genipin. The optimum formulation for the preparation of QCA nanoparticles obtained using BBD was as follows: grafting yield of 50%, QCS/TPP weight ratio of 7.67/1, and QCS/genipin weight ratio of 60/1. The average particle size, PDI, zeta potential, and encapsulation efficiency of the QCA nanoparticles prepared under optimum formulation were found to be 193.68 nm, 0.232, +23.97 mV, and 46.37%, respectively. In vitro BSA release analysis revealed that QCA nanoparticles cross-linked with both TPP and genipin had a much lower initial burst release and a longer release period as compared with the ones cross-linked solely with TPP. According to the results of this study, the QCA nanoparticles cross-linked with both TPP and genipin have a potential as a drug carrier for therapeutic proteins.

## Figures and Tables

**Table 1:** Selection of variables and their levels.

Independent Variables	Symbol	Levels		
		Low (−1)	Medium (0)	High (+1)
Grafting yield (%)	*A*	30	40	50
QCS: TPP (weight ratio)	*B*	5:1	10:1	15:1
QCS: Genipin (weight ratio)	*C*	20:1	40:1	60:1
**Dependent Variables**	**Symbol**	**Constraints**		
Particle size (nm)	*Y*_1_	<200		
PDI	*Y*_2_	<0.3		
Zeta potential (mV)	*Y*_3_			
Encapsulation efficiency (%)	*Y*_4_	Maximize		

**Table 2:** The pH dependence of water solubility of CS and QCS.

Materials	Transmittance (%)	
	pH = 6.0	pH = 7.0
CS	99.0	71.9
QCS-30	97.2	89.0
QCS-40	96.5	92.6
QCS-50	98.6	97.3

**Table 3:** Experimental runs and observed values of responses for BBD.

Run	Variables			Responses			
	Grafting Yield (%)	QCS: TPP (Weight Ratio)	QCS: Genipin (Weight Ratio)	Particle Size (nm)	PDI	Zeta Potential (mV)	Encapsulation Efficiency (%)
1	30	10:1	60:1	227.91	0.229	20.50	40.28
2	30	10:1	20:1	185.12	0.230	17.10	43.66
3	30	5:1	40:1	2101.53	0.571	23.28	51.59
4	30	15:1	40:1	201.88	0.214	20.03	27.65
5	40	10:1	40:1	171.90	0.125	22.45	41.79
6	40	15:1	60:1	179.64	0.211	20.33	23.87
7	40	15:1	20:1	179.25	0.219	17.95	29.76
8	40	5:1	60:1	296.36	0.238	23.78	44.66
9	40	10:1	40:1	181.55	0.225	20.58	39.71
10	40	5:1	20:1	2519.73	0.525	18.74	65.26
11	40	10:1	40:1	181.07	0.209	21.47	44.01
12	50	15:1	40:1	146.65	0.217	23.82	2.23
13	50	10:1	60:1	167.00	0.224	25.16	27.45
14	50	5:1	40:1	190.72	0.226	26.02	67.76
15	50	10:1	20:1	148.48	0.219	18.97	32.02

**Table 4:** Comparison of the experimental and predicted values of the response variables of the QCS nanoparticles prepared under the optimum conditions.

Response Variables	Experimental Values	Predicted Values	Error (%) ^a^
Particle size (nm)	193.68 ± 44.92	199.69	−3.01
PDI	0.232	0.218	+6.42
Zeta potential (mV)	23.97	25.81	−7.13
Encapsulation efficiency (%)	46.37 ± 2.89	41.6	+11.47

^a^ Error (%) = [(experimental value−predicted value)/predicted value]×100%.
